# Tears(1)Readers of the Bistoury are referred to the article on “Laughter” in the number for January, 1883, of which this is meant to be a companion piece.

**Published:** 1885-01

**Authors:** Ausburn Towner


### TEARS. (1)

AUSBURN TOWNER.

There are a number of words in the English language, some of them by their mere sound, clearly expressing the idea that is sought to be conveyed; others whose origin lost in the far past, but connected with the idea within so strongly by ancient and constant usage, that the utterance of them brings up a train of thoughts only allied and associated (2) with them. Most of the first kinds of words alluded to are of home growth, or of Saxon origin, like whirr, for example, which is very plainly the approximate sound made by certain objects in nature or art, expressive to us of the result of a certain action. Or grating, does not that, indeed instantly suggest to us what happens on the movement of a rusty hinge or of teeth rough, hard and uneven?

Many of the other kinds of words mentioned are foreign importations, incorporated into our language from other tongues. Beautiful, for instance, which has been said to be the most beautiful word in the English language. Or home, the liquid semi-vowel dwelling long on our lips, even as the memory of the place designated lingers long in our recollection, wherever our lot may be cast. Or further, the word, tears. A most suggestive term, enveloping within its five letters, volumns of mean-

(1) Readers of the BISTOURY are referred to the article on "Laughter" in the number for January, 1883, of which this is meant to be a companion piece.

(2) It is quite accurate to observe here, that association has much to do with the impression a word when seen or heard makes on our minds, whatever may be its original or inherent meaning. This is especially true as regards proper names, whether Christian or surnames. Everyone of my readers must be able to illustrate this for himself. Take the names of Hannah, Mary, Eliza, for instance, neither of them by their construction or sound seeming very attractive or pretty. But I know those who regard them as being the most beautiful names that could be bestowed on a human being. This, simply, because those they designate possess to them supremely lovable and admirable qualities. So, on the other hand, the names Ethel, Alice, Emeline, that slip so smoothly and sweetly over the tongue and lips, to some, are the reverse of the beauty and charm they seem to possess inherently, and only because they are associated with those possessing to them repulsive or repellant characteristics. In a broader view and more generally, who would think of calling a child Dalilah, Jezebel or Ishmael? Any one of them would seem to carry a curse with it, as they are intimately associated with what is at least unfortunate, although in themselves they are very musical and their meanings not objectionable, the first being "a head of hair," the second only "an isle of habitation" and the third, far from meaning that "his hand is against every man and every man's hand against him," is: "God has heard, or "One whom God hears."

ing. Coupling it with no other word, binding it into no sentence, but allowing it to stand by itself, it appeals to every one with its own sense and meaning, to some in one way, to others, in others, but always in connection with sorrow, unhappiness or misfortune. Tears of joy, tears of gladness and rejoicing, may be heard of and are heard of and experienced, but the term itself must be qualified to take away from it the clear association that belongs to it, and always goes with it, when it stands alone. Tears! Who has not shed them or caused them to be shed? And happy the person who has no recollection of being the occasion of them to those who love him or those whom he loves.

Thoughtfully considered and examined, it is rather peculiar that by tears, a method should be provided by nature as an outward manifestation of emotion. They are practically and honestly for far different purposes, but like many another operation, their primal and everyday work is overlooked and their real use but little considered, being overshadowed by the occasional and infrequent but more open exhibitions they make of themselves in supreme moments of our lives.

All of us who are in a healthy condition, have tears all of the time, although we are not always prepared to shed them, except perhaps some extremely emotional person, who has a reservoir of them constantly on tap and ready to be turned on at the slightest provocation. Some one has recently invented a machine called an "automatic lubricator," out of which he is rapidly making a fortune. It is a little thing and can be attached to any machinery, like a locomotive for instance, and it will keep the joints, axles and every movable piece oiled and in running order all of the time without human help, except to keep its oil reservoir filled. Something of a similar nature every person has attached to the eye or near the eye to keep the eye-lids well lubricated, so that they can act without friction. You rub even as soft and delicate a surface as the inner side of the eye lid, over the equally delicate globe of the eye, as frequently as is done in winking, without some lubricating medium, and the friction would soon be productive of great injury to the sight. This is the office of the tears. They are manufactured in an almond shaped cistern or gland at the inner corners of the eyes and are constantly poured over the eye-balls by means of little pipes or ducts. If the chief use of tears was, what it is not, to supply something with which to weep, we might be said to be crying or manufacturing tears all of the time, that we may have healthy eyes and preserve a good eyesight.

There is no nonsense or mistake in the exclamation or expression, "briny tears," for an analysis of them shows that they contain salt, that is, common table salt, with some albumen and phosphates of lime and soda. These are not the c mponent parts of ordinary oil, but they make up a substance that furnishes the best kind of lubricator for those parts on which they are used.

No one can answer the question why, when one's emotional nature is highly excited, the cistern or gland where the tears are manufactured, becomes so very active and secrets a vastly increased amount of them, sometimes swells up with its exertions and pours them in a flood into the eyes. No one can answer this question, any more than he can the other question why the salivary glands are excited by the touch or approach of some material substance, chewing gum or tobacco, for instance, and set to work instantly to produce additioual quanties of saliva. It can be said that certain nerves inform the brain of the fact that more of the fluid than is on hand is required to dissolve or make away with the intruding matter, and the brain sends back word to go to work and supply the demand. As to the illustration of the salivary glands, the question could be the easier answered than as to the lachrymal or tear-producing glands, for, in the salivary glands, something material is brought into contact with them, and might furnish a portion of the fluid required, or there is something for the fluid to act directly upon. But in the tear-producing gland, the result comes from no material contact. The cause is only a mental one and the manufactory must produce what is required from within, relying not at all upon what is without.

The production of tears, in the case of grief or sorrow, may be likened somewhat, so far as the result produced is concerned, to blushing. A thought or a memory will produce one or the other with as much profussion or intensity, as though the exciting cause was of the present. Why should we weep when we feel bad or are in pain? It cannot be because the sense of sight has control of the matter, although the weeping is produced in the near neighborhood of the organ of such sense. An appeal to any one of our other senses, will often cause the tears to flow, and the blind weep as well as the clear sighted. So far as taste or smell is concerned, we can readily see that some material substance has excited the delicate organs at the upper part of the nose, and nature makes an effort by her water works located there to wash the offending matter away. In the way of feeling, see how quickly the response comes when a foreign substance, be it ever so minute, gets into the eye. The flood gates of the lachrymal gland are instantly and fairly opened to wash away from the organ the uncomfortable intruder. This, not because an appeal has been made to the sense of sight, but to the sense of feeling. Indeed, seeing and hearing are the only two senses an appeal to which, except in intensified cases, has no effect on the tear manufactory.

Any undue physical effort of the eyes, like straining them to look at an object in the distance or in the gloom, will of course start the tears, but that is a peculiarly material view of the case, and as much to be expected and as easy to be accounted for as any other effort of nature to equalize and balance the different parts of the system, as yawning is only an effort of nature to restore the equilibrium of the flexor and extensor muscles.

So the sense of hearing, as simply a sense, never appeals to the tear producer. The sentiment uttered and heard may cause tears to flow and you may have seen persons cry over music that was exquisitely sweet or execrably bad, but in neither of the cases, was it the mere sound produced, that caused the tears.

It is therefore easy to understand why the operation of those senses that excite the production of tears, have the power to do so. But why mere sentiments, emotions or situations have the same power, as has already been said, no one can assuredly and clearly determine. If, as some philosophers assert, ideas are just as material, although not quite as visible, as chunks of chalk or pieces of marble, and always act upon the brain from the outside, making the expression "hit by an idea," a literal and not a figurative one, if this is so, the actual contact of a sentiment or an emotion with the brain might be so unpleasant as to result in a call upon the tears to wash it away. But if this is not so, we can only fall back upon that unsatisfactory but certainly conclusive explanation made use of by Watts in one of his most famous hymns: "It is their nature to."

There are some who have contended that tears and weeping are only artificial, outward expressions of sorrowful feelings as laughter is of joyousness and mirth; that if mankind had only begun in that way, we might now be crying when we would express our gratification, or laughing when we were sad. As though in the early ages, a convention had been held, and after due deliberation, it was decided, that tears should flow in unhappy times, and should be the evidences of grief; and laughter shake its sides when stirred by merriment, and thus it had been ever since. They say it is simply by long association with sadness that tears have come to stand as a sort of representative of that, and the same as to laughter and mirth! What is there in a tear, of itself that indicates sorrow, or in the rising of the midriff that includes a svggestion of mirth? You can often see a tear shining at the end of a man's nose in a cold day, or rolling down his cheek, and they are constantly doing their work, as we have seen, between the globe of the eye and the eyelid. These are pure, genuing tears, unsurrounded by extraneous circumstances and acting the part for which they were intended. Surely, they contain in themselves, no trace of of an element of sorrow or grief. And the hyena and the lunatic go through a physical operation, similar to what is called laughter, and which is indeed pure and simple laughter, yet there is nothing mirthful or amusing in it. Why then, should tears, manufactured and used constantly for a far more necessary purpose, be employed on occasion, in making a material and observable manifestation of mere mental disturbances? Why should they come to be recognized, as they are, and by popular apprehension supposed to be solely and only for the latter purpose, having no other use or existence whatever? There is much reason indeed for supposing that away back in the past, some one who controlled such matters said: "We'll shed tears when we feel bad," and so it has been ever since. That is, at least, as good an explanation of the matter, considering all things, as can be offered by the most patient investigator, or the most thoughtful philosopher. ([3]).

(3) Certainly, there is as much good sense and appositeness in this explanation as there is in the name given to a cer-

Whatever the origin or why their use as manifestations of feeling, these little saline drops have borne a very important part in the history of the world and of individuals, and have been a power that men and nations have vainly fought against, or been overcome only by utterly disregarding them. They are stronger and mightier than their cousins the rain drops, and these, everyone knows, when sufficiently numerous, are considerably beyond the reach of man to stay or subdue. In both cases all that can be done is to wait until the storm is over, and then, as you can, gather up what fragments remain.

In another view, it is strange what a relief a good, generous flow of tears or a "fit" or "spell of crying" will bring to a sorrowing one, especially when it is remembered what the real office of tears is. It would seem from this, that some mistake had been made in locating the seat of the sentiments and emotions, and that it should be in the eye rather than the heart. (4). Certainly when any emotion is to be the most forcibly expressed, so that it may be brought to the apprehension of others, the eye does all of the hardest work and furnishes the most effective weapons.

But withal, it is no wonder that tears, coming to stand, as they do, for sorrow, misfortune and misery, have borne and do bear such a prominent part in the world. It is no hyper bole to call this life "a vale of tears." No person ever lived, who as he approached that other valley where the shadows are so deep and whose opposite side is so dim and uncertain, if not completely blank, could truthfully say that he never had had occasion to shed tears, or who, in balancing up accounts, could aver with accuracy, that he had seen or experienced more in this world that excited his amusement than had aroused his tears. The birth of a child, even amidst the most fortunate surroundings, is rather the subject of apprehension and tears, in the certainty of sorrows disappointments and misfortunes that he must meet with along his journey to the grave, than it is an occasion of rejoicing and glee. And what is that idea that we are only traveling to the grave, but an idea full of tears? A journey with such a termination! How much enjoyment would a traveler take, no matter how beautiful might be the scenery of the countries through which he passes; no matter by how much of delight and pleasure he might be surrounded as he went along, if he knew at the end, and an end to be reached at a moment of which he would have no forewarning, he was to step off a precipice, into the dark and be carried he knew not whither? Yet that is precisely the journey we are all taking! To many, there is a light beyond, which while it robs the hereafter of apprehension does not dry up the tears of the present. Among the promises to them is one that there, whither they are going, there will be no more sorrow or sadness, and it is a curious circumstance that in the Bible wherein are contained all of these promises, the very last time that the word tears is used, as though it would emphasize the fact that they would be done with them forever, is in an assurance that there shall be a place provided where there will be none and no occasion for any. (5).

---

tain kind of wine grown in a certain district in Italy and famous all over the world. This is the "Lachrymæ Christi," or "Tears of Christ." It is a dark, rich wine, and esteemed by some to be more than worth its weight in gold. The grapes from which it is pressed can be grown only in one particular spot in Italy. They are peculiarly flavored, and can be distinguished always by anyone who has once tasted them. The wine is supposed to be the same over which the Latin poet Horace goes into such ecstacies under the name of "Falernian," and wine, the name of which such a toper as Horace has made immortal, must indeed have been a tipple worthy the palate of the gods.

(4) It is rather a cruel misapplication of terms, looked at in this light, that when one would indicate his disbelief in a statement, or show that he was not convinced as to the accuracy of a situation, or manifest an assurance of the insincerity of a companion, the strongest way in which he can put his feelings is in the remark: "It's all in my eye!"

(5.) "For the Lamb * * * * * shall lead them unto living fountains of waters: and God shall wipe all tears from their eyes." Rev. 7:17.

---

CITIES WITHOUT SEWERS.—St. Petersburg, with a population of nearly 1,000,000, and the high death rate of 35.2 per 1,000, is without sewerage, and its water supply is taken from the river Neva, more or less contaminated by percolation from the subsoil. Cairo, with a death rate of 37 per 1,000, is supplied with water from the Nile, having no sewers, and the sewerage filtering through the subsoil into the Nile above the water intake. Vienna, with a death rate of 29 2 per 1,000, has an average of 60 people in each house, or twice as many as in Paris, while the ratable value of the houses in Vienna is only one-sixth more than those in Paris. Pekin, with a death rate of 50 per 1,000, is without proper sewerage, water supply, street cleansing, or other proper sanitary arrangements. The latest scientific systems of sewerage are in use in but few of the great cities."

